# Characteristics of E-Scooter-Related Maxillofacial Injuries over 2019–2022—Retrospective Study from Poznan, Poland

**DOI:** 10.3390/jcm12113690

**Published:** 2023-05-26

**Authors:** Justyna Kowalczewska, Szymon Rzepczyk, Maciej Okła, Kacper Nijakowski, Łukasz Słowik, Aleksandra Makuch, Oliwia Hryniewicz, Julia Krasnoborska, Czesław Żaba, Krzysztof Osmola

**Affiliations:** 1 Department of Neurosurgery and Neurotraumatology, Poznan University of Medical Sciences, Przybyszewskiego 49, 60-355 Poznan, Poland; 2 Department of Forensic Medicine, Poznan University of Medical Sciences, Rokietnicka 10, 60-806 Poznan, Poland; 3 Department of Maxillofacial Surgery, Poznan University of Medical Sciences, Przybyszewskiego 49, 60-355 Poznan, Poland; 4 Department of Conservative Dentistry and Endodontics, Poznan University of Medical Sciences, Bukowska 70, 60-812 Poznan, Poland

**Keywords:** electric scooters, road accidents, facial trauma, maxillofacial injury, traffic safety

## Abstract

Recently, there has been a considerable rise in the popularity and use of electric scooters. Because of this, the number of accidents involving them has also risen. Head and neck injuries are the most common. The aim of the study was to determine the most frequent craniofacial injuries resulting from accidents involving electric scooters, and to identify the risk factors directly related to their placement and severity. The study carried out a retrospective analysis of the medical records of patients of the Clinic of Maxillofacial Surgery over 2019–2022, in terms of craniofacial injuries suffered as a result of e-scooter-related accidents. In the study population (31 cases), of which 61.3% were men, the median age was 27 years. At the time of the accident, 32.3% patients were under the influence of alcohol. Accidents were most common in the 21–30 age group; more often than not, they occurred during warm months and on weekends. The study identified a total of 40 fractures in the patients. The most common craniofacial injuries were mandibular fractures (37.5%), zygomatic-orbital fractures (20%) and frontal bone fractures (10%). A multidimensional correspondence analysis was also performed, which showed that at an age of under 30, alcohol consumption and female gender were associated with a higher likelihood of mandibular fracture. Proper education on the risks associated with the use of e-scooters is essential, with particular emphasis on the impact of alcohol on the driver. It is important to develop diagnostic and therapeutic algorithms for doctors, both in ED and in specialised departments.

## 1. Introduction

The introduction and increase in the popularity of electric scooters have had a significant impact on urban micromobility [1,2]. Low cost, convenience in use and wide availability, thanks to the existence of many scooter rental companies, combined with environmentally friendly transport, are among the main factors that raise the interest and use of electric scooters [3]. They play a particularly important role in large cities, allowing for the avoidance of heavy rush-hour traffic jams, mainly with short-distance travel [4,5].

The first city in Poland to introduce a commercial scooter rental service via a digital application was the capital city, Warsaw, in 2018 [6]. Subsequently, the systems were introduced in other large Polish cities (in Poznan, the introduction of companies allowing e-scooter rental via an application took place in December 2018). Currently, such services are also available in smaller towns. The maximum speed allowed by law for an electric scooter in Poland is 20 kilometres per hour, although commercially sold vehicles can reach much higher speeds [6]. In addition, electric scooters can be used by people from the age of 10, and there is no obligation to wear a helmet.

With the increase in the number of users of electric scooters, the number of accidents involving them has increased significantly [7,8,9,10]. The casualties are most commonly drivers of scooters, who fall as a result of an impact with improper infrastructure or another vehicle [6,11,12]. Because of this, determining the injury pattern and assessing the circumstances of the incidents are necessary. The head is one of the areas of the body that are most vulnerable to injury as a result of an electric scooter accident [13,14]. Research shows that the use of helmets among e-scooter users rarely exceeds 5%; furthermore, most of the helmet models used provide little protection for the facial bones [15].

This study aimed to determine the most common craniofacial injuries resulting from accidents involving electric scooters, and to identify the risk factors directly related to their placement and severity. Casualties’ gender, age and the time of the events were also analysed in order to determine the most usual circumstances of accident. Additionally, the time between the accident and admission to a medical aid point was evaluated, and the duration of the hospitalisation assessed.

## 2. Materials and Methods

The medical records of patients of the Department and Clinic of Maxillofacial Surgery at the University Clinical Hospital of the Poznan University of Medical Sciences from a 4-year period between January 2019 (in Poznan, the introduction of companies allowing for the rental of e-scooters via an application took place in December 2018) and December 2022 were subjected to a retrospective analysis. The criterion for including a case in the research group was for its medical records to contain information about the accident taking place while using an electric scooter. Cases of people who were injured as a result of being hit by an electric scooter were excluded from the study. Cases defined in the documentation as a “traffic accident” without providing the characteristics of the event were also excluded from the study. All patients included in the study required major surgical procedures such as the surgical stabilisation of bone fractures under general anaesthesia. The study did not include injuries without bone damage, such as simple surgical procedures such as suturing under local anaesthetics. Cases meeting the inclusion criteria for the study were analysed in terms of gender, age, alcohol use, the time of the accident, injuries, time between the accident and admission to the hospital, and duration of hospitalisation. The documentation did not record data on the use of a helmet during the accident.

### Statistical Analysis

Epidemiological data on accident frequency are presented in bar graphs. Quantitative variables were compared using the Mann-Whitney test (due to non-compliance with the normal distribution in the Shapiro-Wilk test), and qualitative variables using the Pearson’s Chi-squared test or Fisher’s exact test. Continuous data were presented as medians and quartile ranges. Multidimensional correspondence analysis were used to assess the relationship between the most frequent fractures and the individual factors, such as gender, age and alcohol intake. The significance level was set at α = 0.05 for all analyses. The statistical analysis was performed with Statistica 13.3 (Statsoft, Cracow, Poland).

## 3. Results

### 3.1. Overview

**Figure 1:**
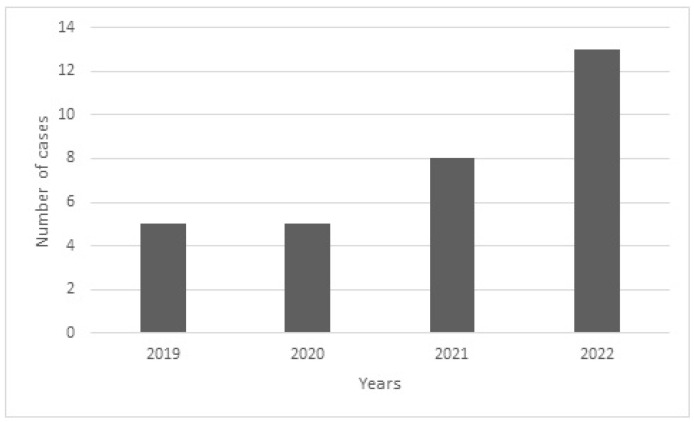
Number of e-scooter-related maxillofacial traumas over 2019–2022.

In the years 2019–2022, 31 cases of accidents on electric scooters were identified in the analysed material that met the criteria for inclusion in the study. In the following years, there was an increase in the number of patients reporting maxillofacial injuries resulting from an electric scooter accident (Figure 1). During the study period, the number of patients requiring surgery and hospitalisation due to e-scooter related injury demonstrated an increasing trend.

### 3.2. Demographic Profile

Among those included in the study, there were 12 women (median age 23.5) and 19 men (median age 31) (Table 1). The youngest hospitalised patient was 9 years old, while the oldest was 49 years old. Two patients under the age of 18 were included in the analysis. In 32.3% of the cases, the drivers were under the influence of alcohol; 60% of those casualties were women. In the analysed population of women, one in two was under the influence of alcohol, while in the population of men, it was one in five (Table 1).

### 3.3. Accident Characteristics 

**Figure 2:**
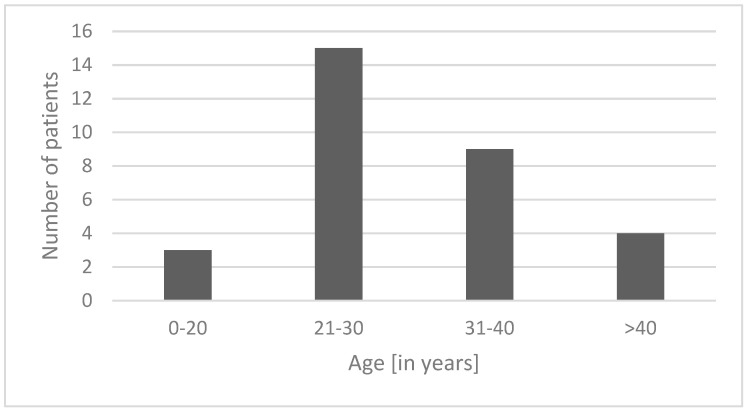
Distribution of patients in age groups.

**Figure 3:**
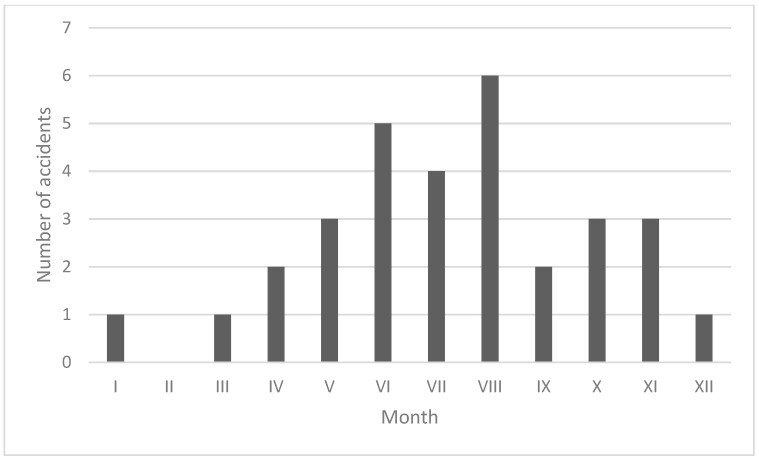
Monthly frequency of e-scooter-related maxillofacial injury over 2019–2022.

Most of the casualties were aged 21–30 (Figure 2). Most e-scooter accidents resulting in craniofacial injuries requiring surgical intervention occurred in the summer months between June and August (Figure 3).

**Figure 4:**
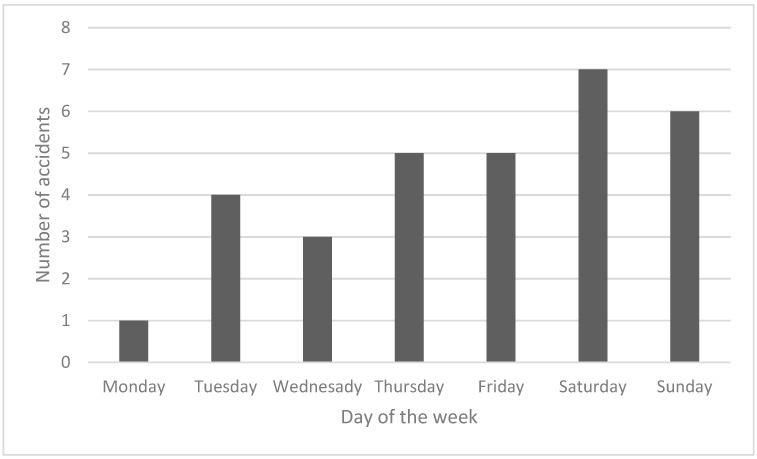
Distribution of accidents involving e-scooters across the week.

An analysis of the cases included in the study showed that almost 42% of accidents involving e-scooters resulting in craniofacial injuries occurred during weekends (Figure 4).

### 3.4. Injury Characteristics

The median time between the accident and the start of hospitalisation was 2 days. There was one case of hospital admission 137 days after the accident. After their accident, the patients spent, on average, 8 days in hospital, though women required significantly longer hospitalisation times (Table 2).

**Figure 5:**
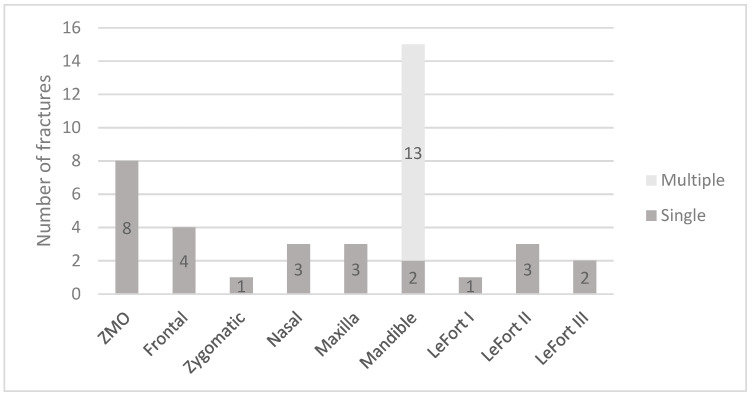
Distribution of craniofacial fractures in the study group (in case of mandible, divided into single and multiple).

In the study group, 40 fractures of the craniofacial bones were recorded (Figure 5). The most common injury was mandibular fracture (15 cases), of which multiple mandibular fractures were much more common than single fractures (13 and 2 cases, respectively). Zygomatic-orbital fractures occurred in 20% of the casualties. In five of the patients, frontal bone fracture was reported. E-scooter related trauma also includes LeFort I (2.5%), LeFort II (7.5%) and LeFort III (5%) type fractures of the maxillary-ethmoidal massive. Three patients experienced nasal bone fracture; in another three patients, maxillary fractures were recognised. An isolated fracture of the zygomatic bone was found in only one patient. In addition, intracranial bleeding occurred in four patients and concussion in three patients.

Moreover, Table 3 presents the distribution of maxillofacial injuries, depending on gender, age and alcohol intake.

### 3.5. Multidimensional Correspondence Analysis

**Figure 6:**
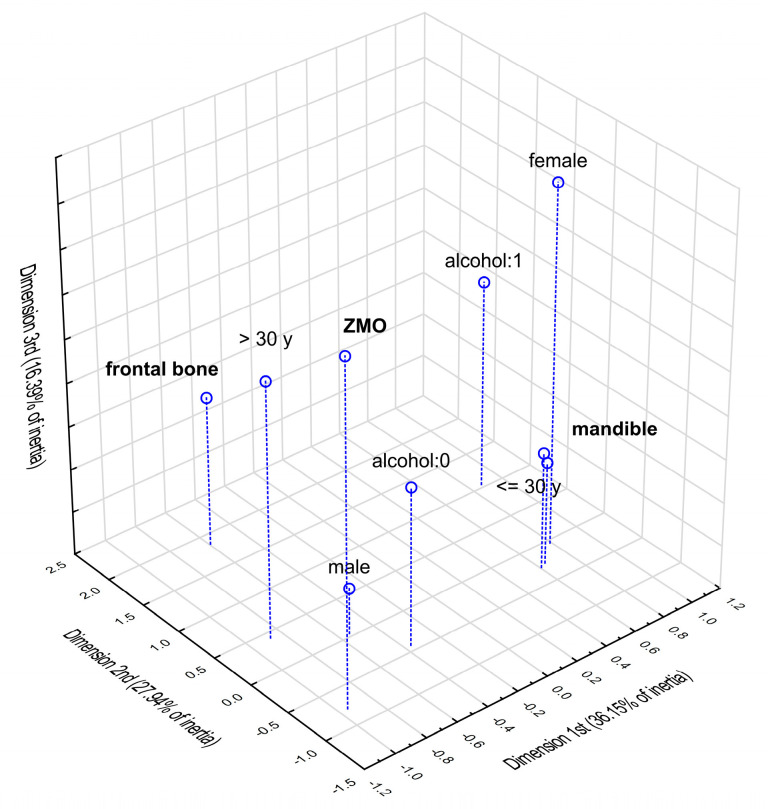
Multidimensional correspondence analysis—3-dimensional plot.

**Figure 7:**
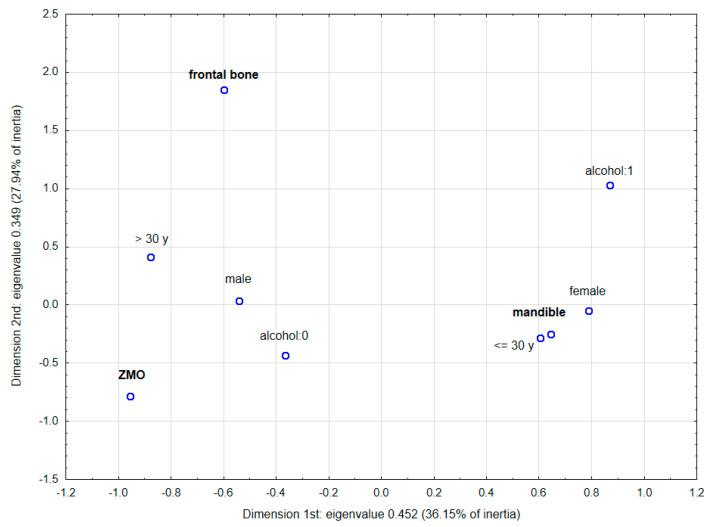
Multidimensional correspondence analysis—2-dimensional plot with the highest inertias.

Multidimensional correspondence analysis (according to Burt’s table 9 × 9) was conducted to graphically present the relationship between the most frequent fractures and the individual factors, such as gender, age and alcohol intake. Based on the scree plot (Figure S1), the three-dimensional analysis was chosen as best describing this association. Figure 6 and Figure 7 show the results of this analysis in three dimensions and in the two dimensions selected with the highest inertias. Parameters characterising the determined points are presented in Table S1. In the case of the three-dimensional plot, the points representing alcohol intake and age below 30 years, as well as female gender, are concentrated closest to mandible, which indicates a higher probability of mandible fractures in these patients. The two-dimensional plot confirms the strongest relationship between an age of below 30 years in females and an increased incidence of mandible fractures (due to the smallest angle with the vertex at the beginning of the coordinate system).

## 4. Discussion

Accidents involving electric scooters most commonly occurred on weekends, during the warm months. The casualties were usually young men. Over 30% of the casualties were under the influence of alcohol at the time of the accident. In the analysed cases, the most frequently damaged craniofacial structure was the mandible. Statistical analysis showed a significant difference in the duration of hospitalisation between women and men. Additionally, a multidimensional correspondence analysis showcased a connection between alcohol consumption, age under 30 and female gender with the risk of mandibular fracture.

As the popularity of e-scooters increases, the number of patients admitting to the emergency department due to injuries sustained during accidents on scooters increases [8,16,17]. Studies show that the number of craniofacial injuries in the US tripled between 2008 and 2017 [18]. An increasing number of patients also require hospitalisation and surgical intervention, which was confirmed in the analysed population—in 2022, the number of patients requiring surgical intervention and hospitalisation in a hospital ward increased 2.6 times compared to 2019. Scooter accidents mostly involve young people [14,19,20]. This is due to the ever-growing popularity of this means of transport resulting from easy access to rental via an application [21,22,23]. 

Studies on injuries related to the use of electric scooters conducted in cities where they are a popular means of transport have shown that the victims of accidents are usually men [24,25,26]. They account for 52% to 79% of victims [7,11,15,16,27,28]. They constituted 61.3% of the studied population. The population of paediatric patients injured as a result of an electric scooter accident is also particularly important [19]. The cases included in the study indicate the possibility of serious injuries resulting from an accident on an electric scooter, which are usually designed exclusively for adults. In an analysis carried out in the USA, the group of patients aged 6–12 was the most affected by accidents on e-scooters; they were involved in as many as 33% of accidents [18]. 

Analyses of the timing of accidents involving electric scooters show that they are clearly more common during the warm months, when this means of transport is most often used, usually between May and August [14]. Similar observations were recognised in the study population, where 58% of accidents occurred in the corresponding period. However, there have been reports of severe craniocerebral injuries sustained during the winter months, in which severe weather-induced road conditions had a clear impact [29,30]. In addition, studies indicate a greater frequency of such events on weekends, usually sustained during returns from social gatherings, at which alcohol is consumed [1,4,14,31]. In addition, accidents occur most often in the afternoon and evening hours [1,11,24]. A study in Finland showed that as many as 74% of such accidents occurred between midnight and 6 A.M. [22]. 

Driving under the influence of alcohol has been identified as a factor which increases the risk of an accident and injury [14,32]. Data from the scoping review show that, on average, about 26.5% of such patients are under the influence of alcohol at the time of injury [13]. In an extreme case, data from studies conducted in Finland indicate that people who sustain injuries while driving scooters are under the influence of alcohol at the time of the event in between 88.9% and 91% of the cases [12,22]. Education on the risks of driving while under the influence of alcohol seems important [33].

The head is the area of the body most vulnerable to injury as a result of an electric scooter accident—injuries to this area of the body occur in 27% to 90% of victims of accidents [7,14,15,34]. Studies show that, at the time of an accident, only 1.3–4.5% of e-scooter drivers usually have a helmet, which is the fundamental element of head protection [13,25,31,35,36]. A study from Israel has shown that wearing a helmet reduces the likelihood of head injury, as well as of hard tissue and dentoalveolar injuries [31]. However, it is worth noting that the most popular models of helmets protecting against brain traumas do not sufficiently protect against all facial injuries.

Studies show that craniofacial injuries most often concern its upper and middle levels, as well as the mandible [13,37,38]. In studies from Korea, the most common craniofacial fracture reported was a nasal fracture, followed by maxillary and mandibular fractures [23]. An analysis from the US also found that the most common fractures in similar cases were nose (27%) and mandibular fractures (10%) [18]. In the analysed population, the mandibular fracture (38.5%) and ZMO fracture (20.5%) were the most common—similar to the study from Helsinki, where 37.8% of patients suffered a mandibular injury and 28.9%, unilateral ZMO fracture [12]. Common injuries that usually do not require surgical intervention include soft tissue injuries overlying the skull, which typically occur in approximately 25% to 70% of casualties [4,7,16,24]. Dental injuries are usually reported in about 25% of victims [12]. 

Among intracranial injuries, the most common are concussion, intracranial hematomas and traumatic brain injury (TBI) [1,39,40]. Serious brain injuries that require neurosurgical intervention are rare, although they can be life-threatening [40,41]. For this reason, it is so important to perform a neurological examination and computed tomography of the head in patients after a scooter accident in order to search for intracranial injuries [14,23,40]. In the analysed population, craniofacial injuries were accompanied by epidural and subdural hematomas in 16% and concussions in 6.5%. 

In addition to craniofacial examination, neurological examination and assessment of possible intracranial injuries, it is also postulated that the abdominal cavities and limbs [14] of patients after injuries on e-scooters should be properly examined, which requires cooperation between specialists from many fields of medicine for the best diagnosis and treatment of this group of patients [15,25]. Deaths from accidents involving electric scooters have also been reported; however, the number of such incidents cannot be properly estimated due to a lack of appropriate studies [2,11,41].

Limitations of the study include the restricted ability to determine the effect of alcohol on injuries resulting from e-scooter accidents. It results from the fact that patients may conceal information about driving under the influence of alcohol, who could not show measurable concentrations of alcohol in the blood when reporting to the hospital after a long time after the accident. Other limitations of the study include a restricted amount of information in the medical records regarding the circumstances, type and time of the accident.

In addition, a limitation of the retrospective study could be the omission or imprecise specification of the type of vehicle in the patient’s documentation, which could have contributed to the omission of some cases.

The sample size is large enough to give an overview of the most common injuries, but not large enough to perform a detailed subgroup analysis examining the risk factors for different types of injuries; therefore, data collection and detailed analyses of larger clinical groups are needed.

## 5. Conclusions

The use of electric scooters may result in serious health consequences, and even death. Therefore, proper education on the risks associated with the use of these vehicles is essential, with particular emphasis on the impact of alcohol on the driver. The authors once again favour the principle of zero alcohol or zero per mile (zero pro-mile or zero per-mile) [1,29]. It is also important to instruct about the significant improvement in safety related to the use of basic protective equipment, such as a helmet, in order to increase the percentage of their use among drivers. An effective solution could be to introduce a legal obligation to wear a helmet while using an e-scooter. An increased age restriction for the use of such vehicles would be recommended in order to avoid injury to the paediatric population when using vehicles inappropriate for their age. It is important to develop diagnostic and therapeutic algorithms for doctors, both in ED and in specialised departments, in order to detect or to rule out the most common dangerous injuries resulting from an accident.

## Figures and Tables

**Table 1:** Participant characteristics.

	Overall *n* = 31	Females *n* = 12	Males *n* = 19	*p*-Value
**Age, years, M** **(Q1–Q3)**	27.0 (22.0–34.0)	23.5 (21.5–31.5)	31.0 (25.0–34.0)	0.193
**Blood alcohol, *n* (%)**	10 (32.3)	6 (50.0)	4 (21.1)	0.127

**Table 2:** Comparison of the time of hospital admission after the accident and the duration of hospitalisation—M (Q1–Q3).

	Overall *n* = 31	Females *n* = 12	Males *n* = 19	*p*-Value
**Admission, days**	2.0 (0.0–6.0)	3.5 (0.5–7.0)	2.0 (0.0–4.0)	0.344
**Hospitalisation, days**	8.0 (5.0–10.0)	9.0 (8.5–11.5)	6.0 (5.0–9.0)	0.013 *

* statistically significant difference.

**Table 3:** Comparison of the distribution of maxillofacial injuries, depending on gender, age and alcohol intake—*n* (%).

Bone Fracture	Gender			Age			Alcohol Intake		
	Female *n* = 14	Male *n* = 26	*p*-Value	≤30 y *n* = 21	>30 y *n* = 19	*p*-Value	Yes *n* = 17	No *n* = 23	*p*-Value
**Frontal**	0 (0.0)	4 (16.0)	0.254	0 (0.0)	4 (22.2)	0.065	2 (11.1)	2 (9.1)	0.033
**Nasal**	1 (6.7)	2 (8.0)		2 (9.0)	1 (5.6)		3 (16.6)	0 (0.0)	
**Zygomatic**	0 (0.0)	1 (4.0)		0 (0.0)	1 (5.6)		1 (5.6)	0 (0.0)	
**ZMO**	3 (20.0)	5 (20.0)		4 (18.1)	4 (22.2)		1 (5.6)	7 (31.8)	
**Maxilla**	1 (6.7)	2 (8.0)		1 (4.6)	2 (11.1)		2 (11.1)	1 (4.6)	
**Mandible**	8 (53.2)	7 (28.0)		12 (54.5)	3 (16.6)		5 (27.7)	10 (45.4)	
**LeFort I**	1 (6.7)	0 (0.0)		1 (4.6)	0 (0.0)		1 (5.6)	0 (0.0)	
**LeFort II**	1 (6.7)	2 (8.0)		1 (4.6)	2 (11.1)		1 (5.6)	2 (9.1)	
**LeFort III**	0 (0.0)	2 (8.0)		1 (4.6)	1 (5.6)		2 (11.1)	0 (0.0)	

## Data Availability

All data supporting the reported results can be found in the archive of the Department of Maxillofacial Surgery of Poznan University of Medical Sciences.

## Supplementary Materials

The following supporting information can be downloaded at: https://www.mdpi.com/article/10.3390/jcm12113690/s1, Figure S1: Scree plot for multidimensional correspondence analysis. Table S1: Multidimensional correspondence analysis—parameters of determined points.
