# Supplementary material for: Characteristics of E-Scooter-Related Maxillofacial Injuries over 2019–2022—Retrospective Study from Poznan, Poland
Source: J Clin Med. 2023 May 26;12(11):3690. doi: 10.3390/jcm12113690 (PMC10253345; doi:10.3390/jcm12113690)
Supplement: Supplementary file 1 [file jcm-12-03690-s001.zip › jcm-2355877-supplementary.pdf]

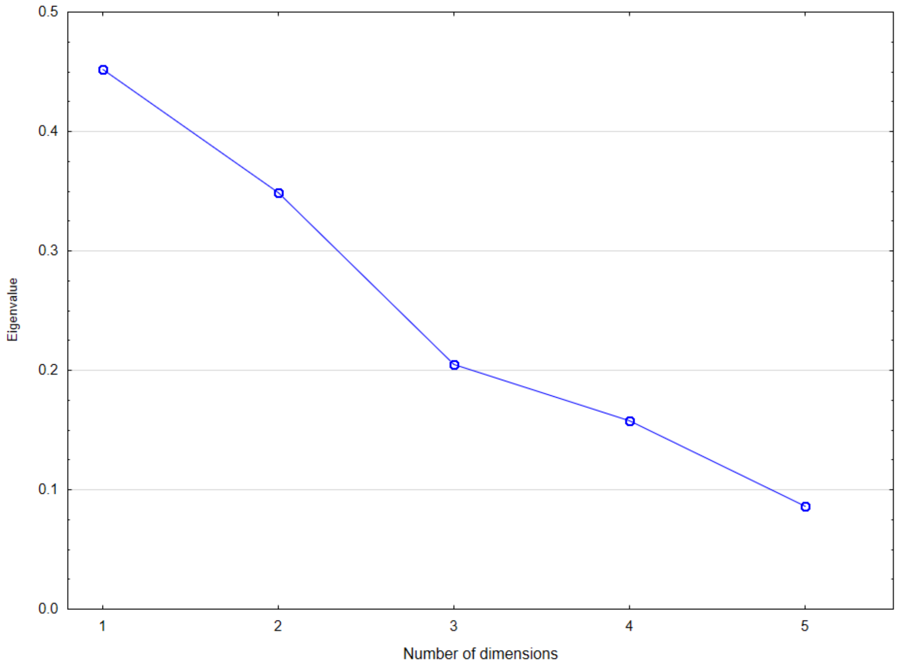

Figure S1: Scree plot for multidimensional correspondence analysis.

Table S1: Multidimensional correspondence analysis—parameters of determined points.

	x	Y	z	Quality	Relative inertia	X		y		z	
						inertia	cos^2	inertia	cos^2	Inertia	cos^2
Gender:male	-0.541	0.033	-0.586	0.927	0.081	0.096	0.426	0.0004	0.002	0.248	0.499
Gender:female	0.788	-0.048	0.852	0.927	0.119	0.140	0.426	0.001	0.002	0.361	0.499
Alcohol:0	-0.366	-0.434	-0.068	0.777	0.059	0.052	0.318	0.095	0.448	0.004	0.011
Alcohol:1	0.869	1.032	0.160	0.777	0.141	0.124	0.318	0.226	0.448	0.009	0.011
Age <= 30 y	0.604	-0.284	-0.258	0.744	0.081	0.119	0.530	0.034	0.117	0.048	0.097
Age > 30 y	-0.878	0.412	0.375	0.744	0.119	0.174	0.530	0.050	0.117	0.070	0.097
Fracture:ZMO	-0.954	-0.782	0.768	0.739	0.148	0.131	0.319	0.113	0.214	0.187	0.207
Fracture:frontal bone	-0.598	1.853	-0.108	0.864	0.163	0.037	0.081	0.455	0.780	0.003	0.003
Fracture:mandible	0.645	-0.253	-0.322	0.729	0.089	0.128	0.519	0.025	0.080	0.070	0.130
